# Publisher’s Note: Continued Publication of *Current Oncology* by MDPI

**DOI:** 10.3390/curroncol28010001

**Published:** 2020-11-20

**Authors:** Delia Mihaila

**Affiliations:** MDPI, St. Alban-Anlage 66, CH-4052 Basel, Switzerland; mihaila@mdpi.com

Current Oncology was launched in 1994 and has published international and multidisciplinary research in the field of cancer therapy, to report on and review progress made in managing this disease over the last 26 years [1]. Current Oncology was published by Multimed Inc. and will be published by MDPI as of January 2021 [2].

Dr. Sharlene Gill from the University of British Columbia has served as Editor-in-Chief of Current Oncology since June 2019 and has kindly agreed to continue to serve in this role. 

We are delighted to take over the publication of Current Oncology, to perpetuate the legacy of this journal and ensure that we serve the scientific community of clinical oncologists in Canada as well as worldwide. While this will continue to be a reference journal for Canadian oncologists, our goal is to greatly expand the journal’s global reach.

Current Oncology complements the MDPI portfolio of journals in this field, especially Cancers [3].

As of 2021, MDPI will continue to publish six bi-monthly issues but aims to increase this frequency to monthly publications in the following years. 

We look forward to publishing your work in Current Oncology! 
